# Government and industry partnership for the development of Dementia Care Competency Standards in the Philippines

**DOI:** 10.1002/alz70858_102545

**Published:** 2025-12-26

**Authors:** Jacqueline C Dominguez, Jiil Arsenal, Kate Marra, Sandie Villaruel, Charline Dominguez, Lavern Ednalan, Roselyn Ramos, Patricia Tayao Mejia, Precy Santiago Cruz, Joel Fornoles, Catherine Horaguchi, Bernadette Audije, IDCA Research Group

**Affiliations:** ^1^ Institute for Dementia Care Asia, Quezon City, Metro Manila, Philippines; ^2^ De Ocampo Memorial College, Manila, Metro Manila, Philippines; ^3^ Technological Education and Skills Development Authority (TESDA), Taguig, Metro Manila, Philippines

## Abstract

Dementia incidence is rising in low‐middle‐income countries (LMIC), majority of economic burden is indirect cost. In the Philippines, informal care by family members is the cornerstone of dementia care, yet programs for dementia education and training are lacking. The Institute for Dementia Care Asia (IDCA) formally initiated a partnership with the Technical Education and Skills Development Authority (TESDA) to create local Dementia Care Competency Standards (CS). Functional analysis was done to define the primary purpose, function, and required skills aligned with industry standards. Key steps were undertaken: a) Industry and Job Analysis to define roles and competencies care workers, focusing on technical, behavioral, and communication skills. b) Development of Units of Competencies (UCs), and c) External Validation.

Twenty industry stakeholders including health and allied health professionals, therapists, educators, environmental design, community workers, and family members with knowledge and experience in dementia care underwent orientation in CS development by TESDA. Since there is a CS on general care for the elderly, skills mapping identified core competencies critical for dementia care which were discussed in bi‐weekly consensus meetings from February to December 2024. Six competencies were derived: 1. Perform Health Assessment on People Living with Dementia (PLwD), 2. Apply Treatment Plan Based on the Determined Level of dementia care 3. Respond to and Manage Behavioral and Psychological Symptoms of Dementia (BPSD), 4. Facilitate Engagement in Recreational and Therapeutic Activities, 5. Facilitate End‐of‐Life Care Planning, and 6. Perform Self‐Care. Descriptors for the work performance such as elements, functions, performance criteria, required knowledge and required skills under each UC were prescribed to ensure standards in training and assessment. The UCs were then presented to experts for external validation. In conclusion, we have created the first Dementia Care Competency Standard in the Philippines. A competency‐based training program will be part of TESDA course offering. Given the constraints of economic its resources but wealth of human resource in the country, this partnership is a big step towards improving dementia care, and the competency standards and qualification is an opportunity for middle‐skilled workers to upskill or reskill for an occupation in the fast‐growing global dementia care industry.

